# Exploring the gut microbiome and serum metabolome interplay in non-functioning pituitary neuroendocrine tumors

**DOI:** 10.3389/fmicb.2025.1541683

**Published:** 2025-04-01

**Authors:** Jifang Liu, Zhang Ye, Yi Zhang, Wan Su, Jie Liu, Tianqi Chen, Yanan Shi, Lulu Liu, Jiao Lu, Zian Cai, Qing Zhong, Pei Wang, Jun Pu, Jinghua Liu, Yuchen Wei, Hui Pan, Huijuan Zhu, Kan Deng, Renzhi Wang, Lin Lu, Xiaomin Hu, Yong Yao

**Affiliations:** ^1^Department of Neurosurgery, Peking Union Medical College Hospital, Chinese Academy of Medical Sciences and Peking Union Medical College, Beijing, China; ^2^Pituitary Disease Innovative Diagnosis and Treatment Center, Peking Union Medical College Hospital, Chinese Academy of Medical Sciences and Peking Union Medical College, Beijing, China; ^3^Department of Endocrinology, Peking Union Medical College Hospital, Chinese Academy of Medical Sciences and Peking Union Medical College, Beijing, China; ^4^State Key Laboratory for Complex Severe and Rare Diseases, Biomedical Engineering Facility of National Infrastructures for Translational Medicine, Peking Union Medical College Hospital, Chinese Academy of Medical Sciences and Peking Union Medical College, Beijing, China; ^5^School of Medicine, Life and Health Sciences, The Chinese University of Hong Kong, Shenzhen, China

**Keywords:** NF-PitNETs, gut microbiota, serum metabolomics, microbiome-metabolome interactions, tumor aggressiveness

## Abstract

The gut microbiome has emerged as a potential factor in cancer pathogenesis, but its role in non-functioning pituitary neuroendocrine tumors (NF-PitNETs) remains unclear. This study aimed to elucidate gut microbiome and metabolomic alterations in NF-PitNETs by comparing microbial diversity, pathogenic bacteria, and serum metabolomic profiles between NF-PitNET patients and healthy controls. The gut microbiome was assessed through 16S rRNA sequencing, while serum metabolomics was analyzed using mass spectrometry. Correlation analyses identified potential links between microbial characteristics and metabolic markers. The results revealed that specific pathogenic bacteria, such as Bacteroides, were significantly enriched in NF-PitNET patients. Multi-omics correlations suggested that altered microbiota might contribute to NF-PitNET pathogenesis by modulating host metabolic pathways. These findings highlight the potential role of gut microbiome dysbiosis and its metabolic effects in NF-PitNET development, offering insights into possible therapeutic and diagnostic targets.

## Highlights

- Identified distinct gut microbiome changes in NF-PitNET patients versus healthy controls.
- Found significant enrichment of pathogenic bacteria, including Bacteroides, in NF-PitNETs.
- Multi-omics correlations link gut microbiota alterations with NF-PitNET metabolic pathways.

## 1 Introduction

Pituitary neuroendocrine tumors (PitNETs), formerly known as pituitary adenomas(PA), represent the third most common type of brain tumor in adults, comprising approximately 15% of all adult brain tumors (Miller et al., 2021; Ostrom et al., 2021; Melmed, 2020). These tumors are classified based on their hormonal activity into functional and non-functional types. Functional PitNETs often lead to overproduction of hormones, causing conditions such as acromegaly, Cushing’s disease and prolactinoma (Tritos and Miller, 2023; Mehta and Lonser, 2017). Non-functional PitNETs (NF-PitNETs), which make up about 30% of these tumors (Daly and Beckers, 2020), do not secrete hormones and typically present with symptoms like headaches, visual field defects, and pituitary dysfunction due to the physical impact of the tumor on surrounding vital tissue (Tritos and Miller, 2023). Although many PitNETs can be effectively managed with surgical intervention, some display refractory or aggressive behaviors that complicate treatment (Kolitz and Greenman, 2023; Di Ieva et al., 2014; Whitelaw, 2019). Understanding the factors that influence the development and progression of PitNETs is crucial for improving diagnostic and therapeutic approaches for this diverse group of tumors.

The human intestinal microbiota, a complex ecosystem of bacteria, fungi, viruses, archaea, and other microorganisms, forms symbiotic relationships with the host that significantly impact various aspects of health (Sender et al., 2016; Kahrstrom et al., 2016). Previous studies have demonstrated a significant correlation between intestinal dysbiosis and the prevalence of neurological diseases (Aho et al., 2021), cancer (Helmink et al., 2019), gastrointestinal disorders (Jones and Neish, 2021), cardiovascular diseases (Zhu et al., 2023), and other conditions (Lou et al., 2022). Extensive experimental data and epidemiological evidence further indicate that an imbalance in intestinal flora plays a key role in the progression of various cancers, including breast, lung, colorectal, prostate, gastric, and liver cancer (Liu et al., 2024). Alterations in the composition of the intestinal microbiota and the modulation of associated metabolites have the potential to regulate cellular metabolism and immune function, establishing a scientifically sound connection between intestinal flora and cancer development (Nandi et al., 2023; Li et al., 2024). For instance, deoxycholic acid (DCA), produced by *Clostridium*, can cause DNA damage through enterohepatic circulation and induce a senescence-associated secretory phenotype (SASP) in hepatic stellate cells. This process is accompanied by the release of various inflammatory cytokines and growth factors, which may promote the development of inflammation-associated, obesity-related hepatocellular carcinoma (Yoshimoto et al., 2013). Similarly, *Bacteroides fragilis*, through *B. fragilis* toxin (BFT), not only accelerates tumor growth and metastasis but also significantly enhances the self-renewal ability of breast cancer cells by concurrently activating the *β*-catenin and NOTCH1 signaling pathways, providing new opportunities for tumor progression (Parida et al., 2021).

Despite significant advances, research on the gut microbiome’s role in PitNETs is limited. Existing studies indicate notable differences in the microbial compositions of patients with pituitary somatotroph tumors and NF-PitNETs compared to healthy individuals (Lin et al., 2022; Hu et al., 2022; Hacioglu et al., 2021; Sahin et al., 2022; Nie et al., 2022). However, these observations primarily focus on microbial characteristics at a single omics level, limiting a comprehensive understanding of the pathophysiological mechanisms underlying PitNETs. A deeper exploration of the systemic biological changes within the PitNETs microbiome and serum metabolome is essential to unravel the complex interplay between the gut microbiota and the development and progression of PitNETs.

This study recruited 23 patients with NF-PitNETs and 30 healthy controls (HC). By utilizing 16S rRNA gene amplicon sequencing and non-targeted metabolomics to analyze fecal and serum samples, we aimed to study the diversity and abundance of fecal microorganisms and serum metabolites in NF-PitNET patients, as well as their relationship with the clinical characteristics of PitNETs. This study provides new insights into the complex interactions between the gut microbiota and the development and progression of PitNETs.

## 2 Materials and methods

### 2.1 Ethical statement

This study received approval from the Ethics Committee of Peking Union Medical College Hospital (Reference: K5112), adhering strictly to ethical standards. In line with the principle of informed consent, all participants provided written consent after being fully informed about the study’s purpose and procedures.

### 2.2 Participants

Patients newly diagnosed with NF-PitNETs and treated surgically were recruited from the Department of Neurosurgery, Peking Union Medical College Hospital, between December 2022 and March 2023. The diagnosis for all specimens was confirmed through pathological examination. Additionally, 30 healthy individuals from the eligible population in Beijing, China, were enrolled as controls. To minimize potential confounding factors, the following exclusion criteria were applied to both groups: (1) antibiotic and/or probiotic use within the past 6 months; (2) history of chronic gastrointestinal diseases or surgeries within the past year; (3) presence of malignant tumors, autoimmune diseases, or infectious diseases; and (4) significant dietary changes or new dietary supplements affecting intestinal flora within the past 3 months. Comprehensive clinical data were collected, including demographic characteristics (age, gender, height, weight, and body mass index [BMI]); clinical features (symptoms and duration of illness); tumor characteristics (size, Knosp grade, and aggressiveness); and pathological parameters (Ki67 and P53 levels).

### 2.3 Sample collection

Fresh stool samples from each participant were collected, divided into two 50 mg portions, and stored in sterile cryogenic tubes. The tubes were immediately placed in ice boxes and transported to the laboratory, where they were stored at −80°C. Serum samples were obtained in the morning following an overnight fast of at least 8 h. Blood samples were collected into vacuum tubes; however, 13 of the 30 healthy control participants declined blood sampling. After collection, the tubes were gently inverted to ensure proper mixing and centrifuged at 3000 rpm for 10 min at 4°C. The resulting supernatant (serum) was transferred into 1.5 mL cryovials and stored at −80°C for future analysis. For NF-PitNET patients, all sample collections were completed prior to surgical intervention.

### 2.4 16S rRNA gene amplicon sequencing and analysis

Genomic DNA was extracted from fecal samples by Novogene Biotechnology Co., Ltd. The V4 region of the 16S rRNA gene was amplified using specific primers. After library construction, DNA quantification was performed using Qubit and qPCR, followed by PE250 sequencing on the NovaSeq 6000 platform. Sequencing data were demultiplexed based on barcode and PCR primer sequences. After trimming barcode and primer sequences, FLASH (Version 1.2.11) was used to merge paired-end reads, generating raw tag sequences (Raw Tags). Cutadapt was used to remove residual primer sequences to avoid interference in downstream analyses. Fastp (Version 0.23.1) was applied to filter low-quality sequences, producing high-quality tags (Clean Tags). Chimeric sequences were identified and removed by comparing tag sequences against the SILVA database,^1^ resulting in high-quality effective tags (Effective Tags). The UPARSE algorithm (Version 7.0.1001) was used to cluster effective tags from all samples into operational taxonomic units (OTUs) at a 97% similarity threshold. Representative OTU sequences were selected based on the highest frequency within each cluster. Taxonomic classification was performed using the SILVA database and the Mothur algorithm.

Alpha diversity (Shannon and Simpson indices) and beta diversity (weighted UniFrac) were calculated using QIIME (Version 1.9.1) to assess microbial diversity. Principal coordinate analysis (PCoA) was performed and visualized using the ade4 and ggplot2 packages in R. ANOSIM, Adonis, and MRPP tests were applied to evaluate differences between groups. Rarefaction curves were generated in R using the plyr package.Venn diagrams were generated in R using the VennDiagram package. The top 10 most abundant taxa at different taxonomic levels (phylum and genus) were visualized using distribution histograms and chord diagrams, generated in Perl with the SVG function. PICRUSt2 (Version 2.3.0) was used to predict microbial functional profiles by normalizing 16S rRNA data, estimating gene family abundance, and mapping functional pathways to KEGG Orthologs. Functional differences between NF-PitNET patients and healthy controls were analyzed using the Wilcoxon rank-sum test, with FDR correction applied to adjust for multiple comparisons. BugBase was employed to predict the relative abundance of potentially pathogenic bacteria, and statistical differences between groups were assessed using the Wilcoxon rank-sum test with FDR correction. LEfSe was used to identify differentially abundant bacterial taxa between NF-PitNET patients and healthy controls through a three-step process: Kruskal-Wallis test for feature selection, Wilcoxon rank-sum test for pairwise comparisons, and Linear Discriminant Analysis (LDA) to estimate effect sizes, with results visualized using LDA score plots and cladograms.

Differentially abundant bacterial taxa were analyzed using DESeq2, applying a negative binomial GLM to estimate log2 fold changes and *p*-values. A volcano plot was constructed to visualize significant taxa, with the x-axis representing log2 fold changes and the y-axis showing the -log10 p-values. Bacterial abundance differences were compared using violin plots, with Wilcoxon rank-sum determining statistical significance.

### 2.5 LC/MS non-targeted metabolomics analysis

This study employs liquid chromatography-mass spectrometry (LC–MS) for non-targeted metabolomics analysis. Raw data were preprocessed using Compound Discoverer 3.3 (CD3.3, Thermo Fisher Scientific, United States). Initial data screening was conducted based on retention time and mass-to-charge ratio (m/z), followed by peak alignment across different samples to enhance identification accuracy. Peaks were extracted based on predefined ppm thresholds and adduct ion information, with simultaneous quantification of peak areas.Metabolite identification was performed by comparing experimental spectra against high-resolution spectral databases (mzCloud, mzVault, and MassList). The molecular weight of each metabolite was determined based on the m/z ratio of the parent ion in the primary mass spectrum, and the molecular formula was predicted using mass deviation (ppm) and adduct ion patterns before matching with reference databases. Secondary metabolite identification was conducted by matching fragment ions, collision energies, and other spectral parameters with database records.Metabolites with a coefficient of variation (CV) <30% in quality control (QC) samples were retained for subsequent analysis. Chromatographic peaks were integrated using CD3.3, with the peak area of each characteristic peak representing the relative abundance of the corresponding metabolite. Metabolite quantification was standardized using total peak area normalization to ensure data comparability.

Principal component analysis (PCA) was performed on the extracted peak data, and logarithmic transformation and standardization were conducted using MetaX software.To investigate metabolic and microbial differences between NF-PitNET patients and healthy controls, various statistical and visualization approaches were applied. A heatmap was generated using the pheatmap package in R to display the relative abundance of metabolites, with hierarchical clustering performed using Euclidean distance and Ward’s linkage method. Differential metabolite analysis was conducted using the Wilcoxon rank-sum test, and a volcano plot was created with the ggplot2 package to highlight significantly altered metabolites (|log2 fold change| > 1, *p* < 0.05). KEGG pathway enrichment analysis was performed using MetaboAnalyst 5.0, with significant pathways (*p* < 0.05) visualized using bubble plots.A violin plot was used to compare key differential metabolite distributions, with statistical significance assessed by the Wilcoxon rank-sum test.

### 2.6 Multi-omics correlation analyses

Spearman correlations between important bacterial taxa, serum metabolites and clinical parameters were calculated in SPSS software. Correlations between features were visualized using the pheatmap package.

### 2.7 Statistical analysis

Descriptive statistics, such as means, medians, and standard deviations, were utilized to encapsulate the study population’s characteristics. The normality of data distributions was evaluated using the Shapiro–Wilk test. For normally distributed continuous variables, t-tests were applied to determine statistical differences. The Mann–Whitney U test was employed for continuous variables not following a normal distribution. Categorical variables were analyzed using either the chi-square test or Fisher’s exact test, depending on the data’s suitability. Correlation between variables was assessed through Spearman’s rank correlation analysis. In all statistical evaluations, a *p*-value below 0.05 was deemed to indicate statistical significance. The analyses were performed using SPSS software version 26.0.

## 3 Results

### 3.1 Study population characteristics

This study included 23 patients with confirmed NF-PitNETs and 30 healthy individuals as controls. Table 1 summarizes the demographic and clinical characteristics of all participants. The analysis revealed no significant differences in gender distribution, age, or BMI between the two groups (Supplementary Tables 1, 2).

**Table 1:** Characteristics of the study cohort.

Characteristic	NF-PitNETs (*n* = 23)	HC (*n* = 30)	*p*- value
Male/Female	12/11	8/22	0.058
Age (years)	57.5 ± 11.8	53.6 ± 9.1	0.175
BMI (kg/m2)	24.8 ± 2.6	23.4 ± 2.6	0.055
Symptom			
Headache	10 (43.5%)	NA	NA
Visual field defects	12 (52.2%)	NA	NA
Pituitary dysfunction	3 (13.0%)	NA	NA
Tumor size(cm^3^)	11.3 ± 11.2	NA	NA
Knosp grade			
I-II	10 (43.5%)	NA	NA
III-IV	13 (56.5%)	NA	NA
Aggressive	14 (60.9%)	NA	NA
Pituitary apoplexy	8 (34.8%)	NA	NA
Pathological characteristics			
**Ki67**			
<3%	12(52.2%)	NA	NA
≥3%	11(47.8%)	NA	NA
P53(+)	6(26.1%)	NA	NA

Quantitative data are presented as mean ± standard deviation. Categorical data are presented as frequency and percentage. NA, Not available.

### 3.2 Overview of gut microbiome across different groups

To compare the gut microbiota composition between patients with NF-PitNETs and healthy controls, we analyzed stool samples from 53 participants using high-throughput sequencing, specifically targeting the V3-V4 region of the 16S rRNA gene. The analysis generated 4,087,091 high-quality 16S rRNA sequences, with a median read count of 77,432 per sample (range: 63,321 to 88,463). After denoising, we identified 1,461 OTUs. Rarefaction curves confirmed that the sequencing depth was adequate (Supplementary Figure S1A).

**Figure 1:**
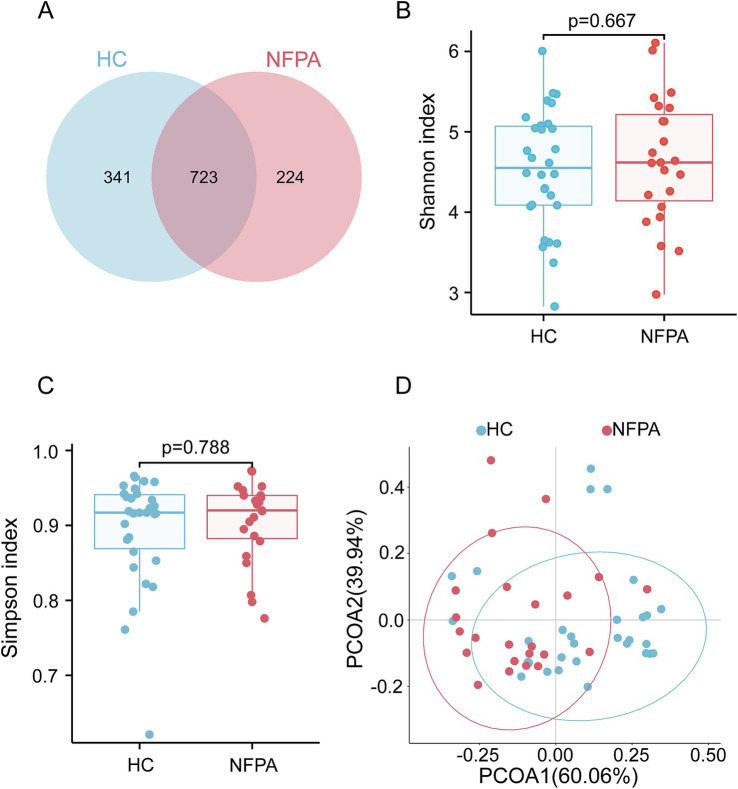
Diversity analysis of the gut microbiota. **(A)** A Venn diagram showing the unique and common OTUs in each group. **(B)** The Shannon index and **(C)** the Simpson index were used to estimate alpha diversity differences between the two groups. **(D)** Beta diversity analysis comparing the two groups.

Of the identified OTUs, 723 were common to both groups, while 341 were unique to the HC group and 224 were unique to the NF-PitNET group (Figure 1A). Analysis of bacterial community composition showed no significant differences in gastrointestinal microbial diversity between the two groups, based on the Shannon and Simpson indices (Figures 1B,C), indicating comparable alpha diversity, with no significant differences in species richness or evenness of the intestinal microbiota.

However, further investigation using weighted UniFrac distances and principal coordinate analysis (PCoA) revealed significant differences in beta diversity between the two cohorts (Figure 1D). These structural differences were statistically significant, as confirmed by Anosim analysis (*R* = 0.174, *p* = 0.001), Adonis analysis (*R*^2^ = 0.110, *p* = 0.001), and MRPP analysis (*p* = 0.001; Supplementary Figure S1B). These findings suggest that the differences between the gut microbiota of NF-PitNET patients and healthy controls are greater than the variations within each group.

### 3.3 Alterations in fecal microbiota composition associated with NF-PitNETs

**Figure 2:**
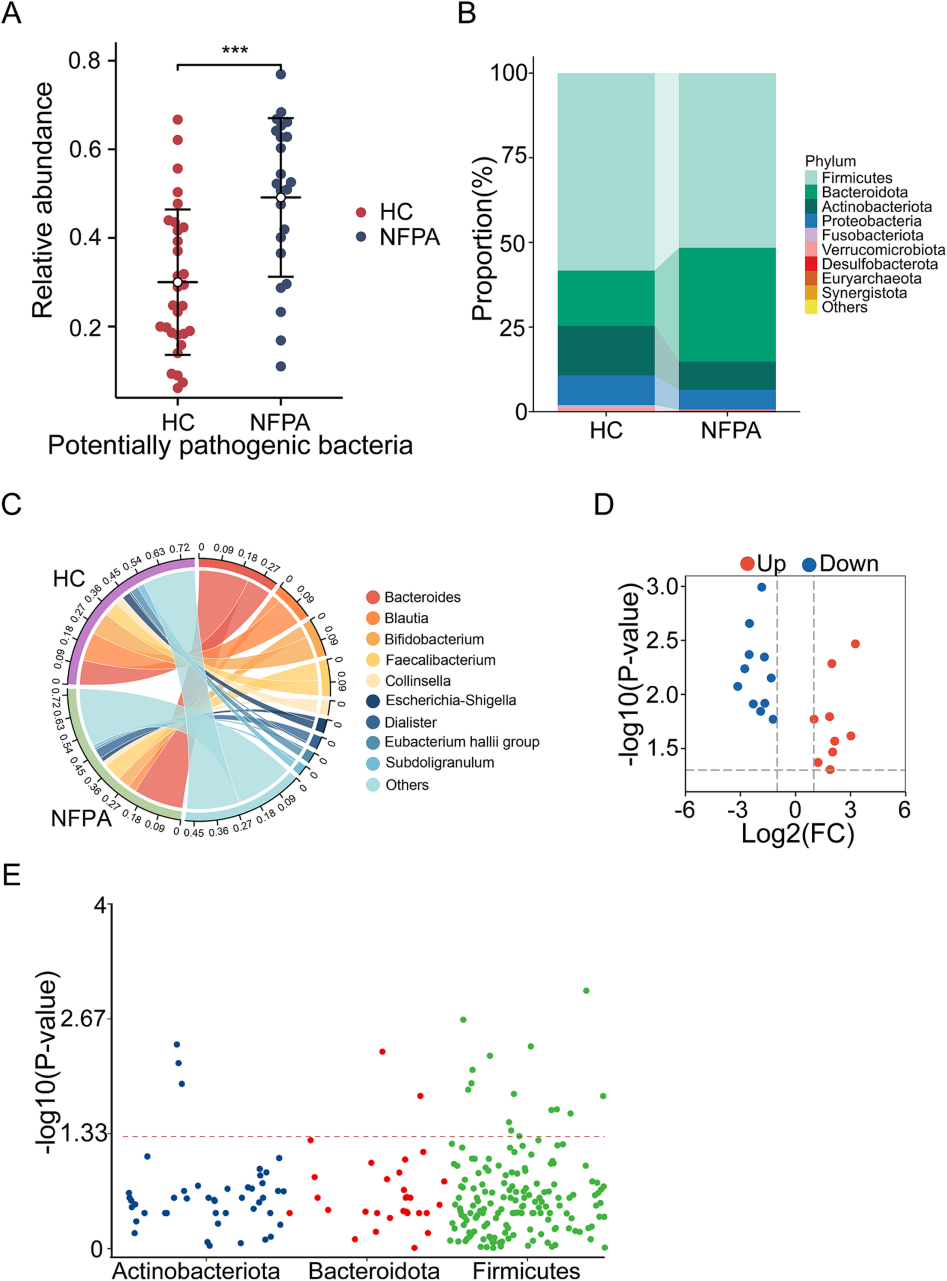
Compositional differences in the gut microbiota between NF-PitNETs and HC groups. **(A)** Relative abundance of potentially pathogenic bacteria predicted using the BugBase database. ***p < 0.001, Mann–Whitney U test. **(B)** Dominant phyla in each group. **(C)** Dominant genera and their relative contributions to each group. **(D)** Volcano plot showing signature bacteria distinguishing the HC and NF-PitNETs groups. **(E)** Distribution of differential genera at the phylum level.

Using the BugBase database, we predicted the phenotypic profiles of the gut flora in each group and found that the aggregate count of potentially pathogenic bacteria was significantly higher in the NF-PitNET group compared to the control group (*p* = 0.0003; Figure 2A).

The relative proportions of the dominant bacterial communities at both the phylum and genus levels were assessed. At the phylum level, Firmicutes and Bacteroidetes were predominant, with Firmicutes representing 58.3% of the microbiota in the control group and 52.6% in the NF-PitNET group, while Bacteroidetes accounted for 16.4% in the control group and increased to 33.6% in the NF-PitNET group. Other notable phyla included Actinobacteria, Proteobacteria, and Fusobacteriota (Figure 2B). At the genus level, the NF-PitNET group exhibited a distinct microbial composition, with Bacteroides (21.8%), Faecalibacterium (7.9%), and Bifidobacterium (5.4%) being predominant. In contrast, the most abundant genera in the control group were Blautia (12.5%), Bacteroides (10.8%), and Bifidobacterium (9.9%) (Figure 2C).

As illustrated in the volcano plot (Figure 2D), a total of 20 genera exhibited significantly different abundances between the two groups, with 9 genera upregulated and 11 genera downregulated in the NF-PitNET group. The Manhattan plot (Figure 2E) depicts the distribution of these 20 differential genera across the three most abundant phyla: Firmicutes, Bacteroidetes, and Actinobacteria.

Linear discriminant analysis (LDA) combined with effect size (LEfSe) identified 36 taxa that were significantly different in abundance between the groups, with 18 taxa dominant in NF-PitNET patients and 18 dominant in healthy controls. Among them, Bacteroidetes (LDA score 4.78, *p* = 0.001) and Parabacteroides (LDA score 3.97, *p* < 0.001) were the most significantly upregulated species with the highest LDA scores in NF-PitNET patients (Supplementary Figures S2A,B).

**Figure 3:**
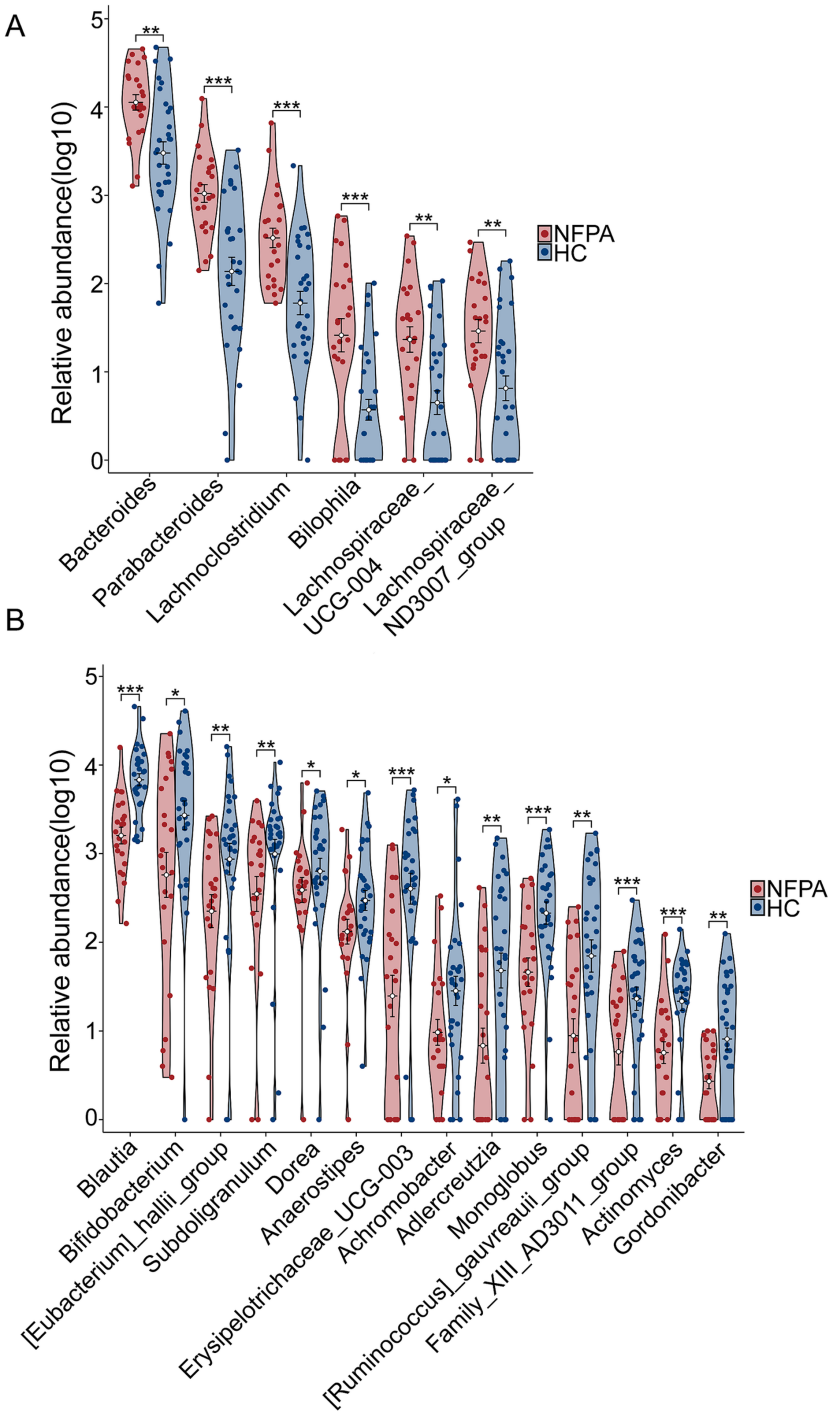
Upregulated and downregulated genera in the NF-PitNETs group. **(A)** Six upregulated genera and **(B)** 14 downregulated genera in the NF-PitNETs group. *p < 0.05, **p < 0.01, ***p < 0.001.

By combining the 20 differential genera identified in the volcano plot with those having LDA scores greater than 3 in LEfSe (Supplementary Figure S3), and excluding genera with an abundance of 0 in too many samples, we identified 6 upregulated (Figure 3A) and 14 downregulated genera (Figure 3B) in NF-PitNETs.

**Figure 4:**
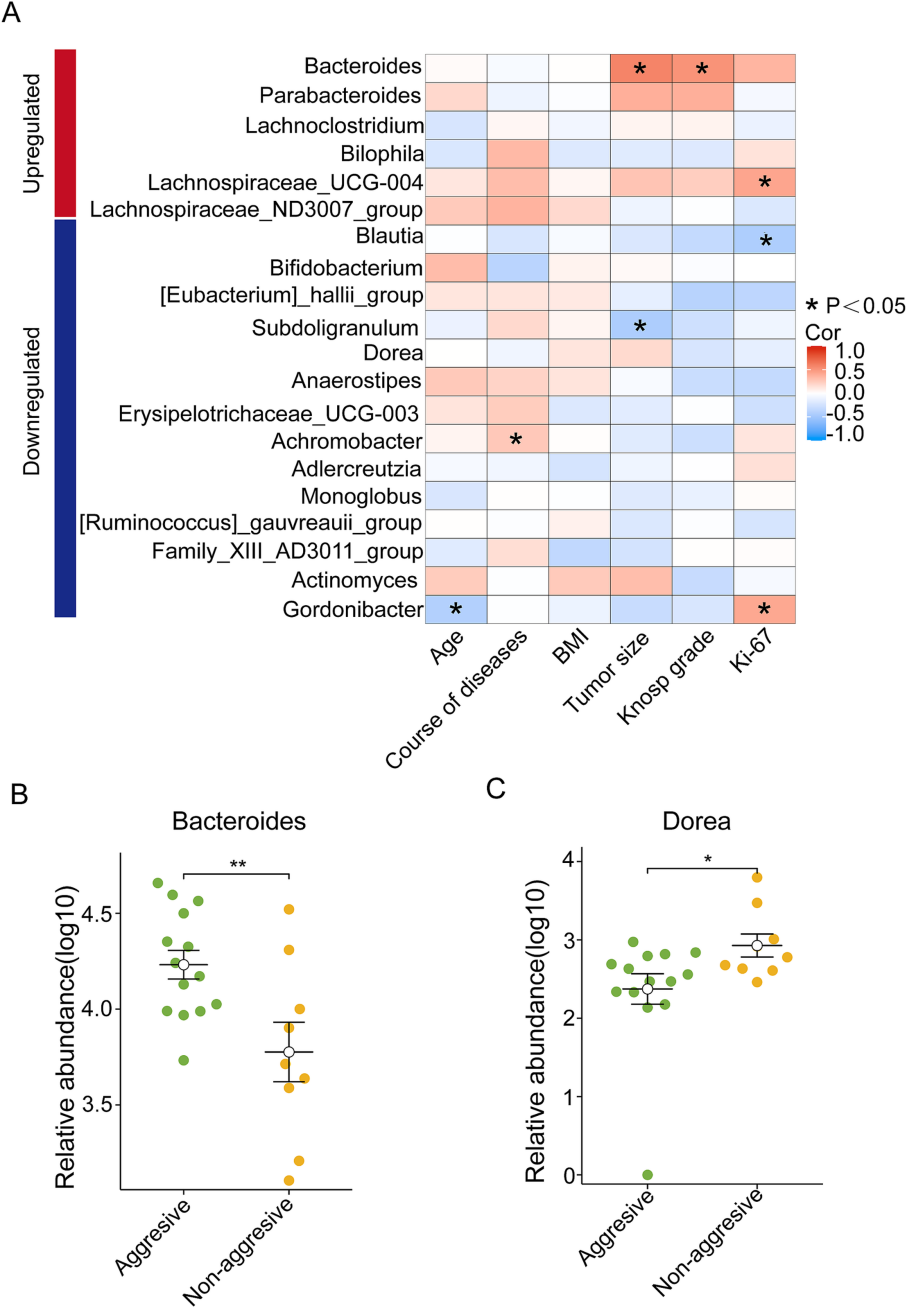
Flora-clinical correlation analysis. **(A)** The upregulated and downregulated bacterial genera in the NF-PitNETs group were associated with the severity of clinical symptoms in patients. *p < 0.05. **(B)** Difference in the abundance of Bacteroides between aggressive and non- aggressive NF-PitNETs. **p < 0.01. **(C)** Difference in the abundance of Dorea between aggressive and non- aggressive NF-PitNETs. *p < 0.05.

To explore the potential relationship between intestinal microbial composition and the clinical characteristics of NF-PitNET patients, a Spearman correlation analysis was conducted on 20 differential bacterial genera in relation to tumor size, Knosp grade, and Ki-67 expression. Notably, among the upregulated genera in NF-PitNETs, Bacteroides showed a positive correlation with tumor size and Knosp grade, while Lachnospiraceae_UCG-004 was positively correlated with Ki-67 expression. Among the downregulated genera, Blautia was negatively correlated with Ki-67 expression, and Subdoligranulum was negatively correlated with tumor size (Figure 4A).

The tumors were further divided into aggressive and non-aggressive groups, and the 20 differential bacterial genera were compared between these two groups (Supplementary Figures S4A,B). The results indicated that Bacteroides had significantly higher expression in the aggressive group compared to the non-aggressive group (Figure 4B), while Dorea, a downregulated genus, showed higher expression in the non-aggressive group than in the aggressive group (Figure 4C).

To investigate the functional contributions of gut microbiota in NF-PitNET patients, we performed PICRUSt2-based predictions using 16S rRNA sequencing data. A total of 96 microbial pathways exhibited significant differences in abundance between the NF-PitNET and HC groups (*p* < 0.05, FDR < 0.2; Supplementary Figure S5), with 52 pathways enriched in the NF-PitNET group. The predicted functional shifts primarily involved carbohydrate metabolism, amino acid metabolism, nucleotide biosynthesis, lipid metabolism, and polyamine biosynthesis, suggesting potential alterations in microbial metabolic activity. Among the carbohydrate metabolism pathways, the superpathway of glycolysis and the Entner-Doudoroff pathway, gluconeogenesis I, and the superpathway of glucose and xylose degradation were significantly enriched in NF-PitNET patients, indicating potential microbial contributions to altered glucose utilization. Additionally, several amino acid metabolism pathways were highly expressed, including phenylalanine, tyrosine, and tryptophan metabolism, L-arginine biosynthesis III, L-histidine degradation I, the superpathway of arginine and polyamine biosynthesis, and the superpathway of polyamine biosynthesis I & II, reflecting microbial involvement in nitrogen metabolism. Furthermore, multiple nucleotide biosynthesis pathways were enriched, including the superpathway of purine nucleotides *de novo* biosynthesis I & II, superpathway of guanosine nucleotides de novo biosynthesis I & II, and superpathway of pyrimidine ribonucleotides de novo biosynthesis, indicating increased microbial potential for purine and pyrimidine synthesis. Notably, lipid metabolism pathways, such as fatty acid elongation – saturated, lipid IVA biosynthesis (*E. coli*), and Kdo transfer to lipid IVA, were also significantly elevated in the NF-PitNET group, suggesting shifts in microbial lipid processing.

The Shannon and Simpson indices were used to evaluate the aggressive and non-aggressive groups. Bacterial community composition analysis revealed no significant difference in gastrointestinal microbial diversity between the two groups (Supplementary Figure S6A). Similarly, beta diversity analysis using weighted UniFrac distance and principal coordinate analysis (PCoA) showed no statistically significant differences in microbial community structure between aggressive and non- aggressive tumors (Supplementary Figure S6B).

Linear discriminant analysis (LDA) effect size (LEfSe) identified 42 taxa with significantly different abundances between the two groups, with 14 taxa enriched in the aggressive tumor group and 28 taxa enriched in the non- aggressive tumor group. Notably, Bacteroides was significantly upregulated in the aggressive tumor group (LDA score = 4.91, *p* = 0.008), whereas Dorea was significantly downregulated (LDA score = 3.86, *p* = 0.03). These findings were consistent with prior analyses (Supplementary Figure S7), further supporting the distinct microbial composition associated with tumor invasion.

### 3.4 Alterations in plasma metabolite profiles in NF-PitNET patients and identification of key metabolites

**Figure 5:**
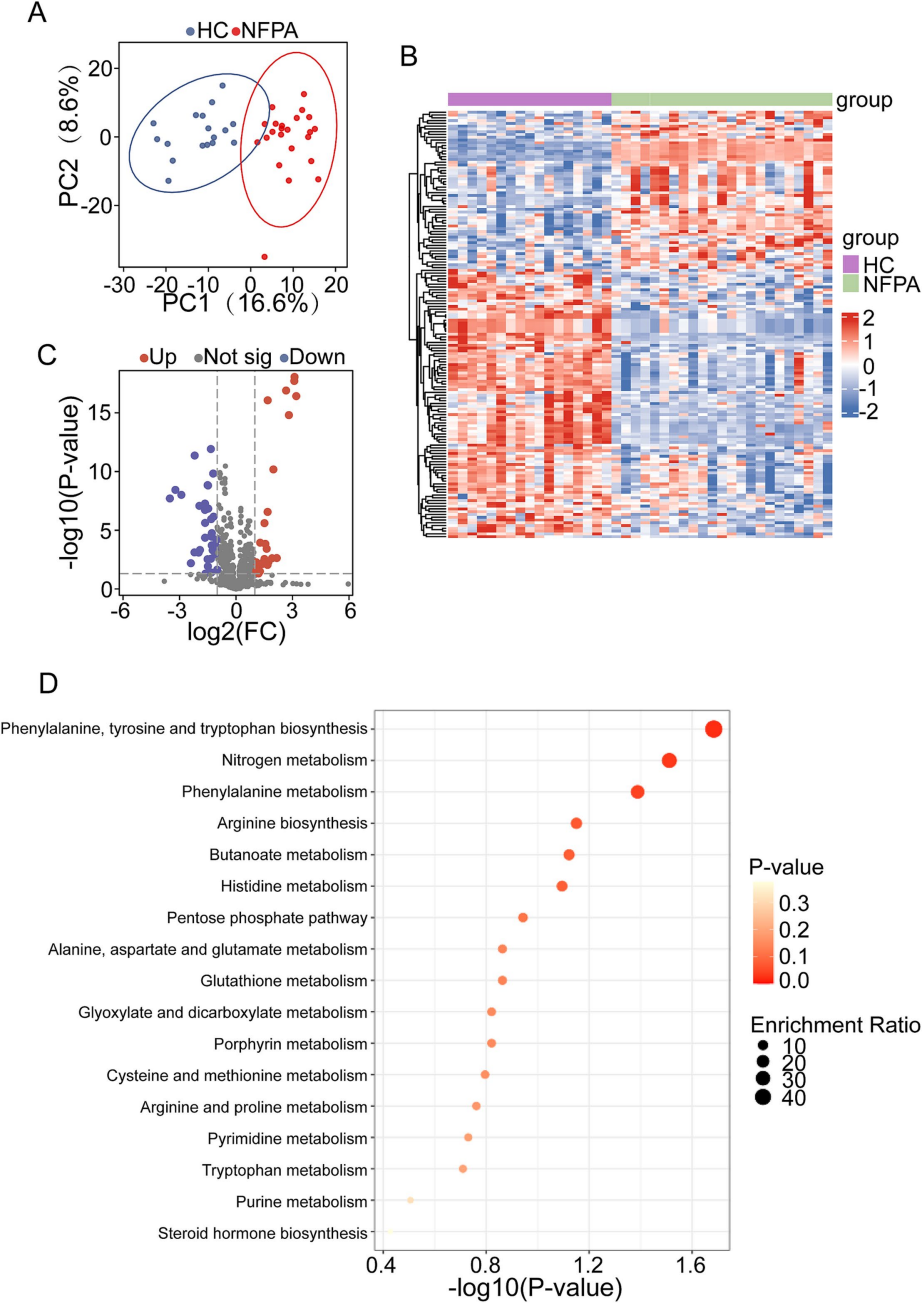
Changes in the plasma metabolite profile in NF-PitNETs patients. **(A)** PCA revealed significant differences in serum metabolic profiles between the NF-PitNETs and HC groups. **(B)** Heatmap of differential metabolites in NF-PitNETs and HC groups. **(C)** Volcano plot of differentially expressed serum metabolites. **(D)** KEGG pathway enrichment analysis of differential metabolites.

Metabolites and fermentation products produced by the intestinal flora can enter the bloodstream and significantly influence host physiological functions. To further investigate these microbe-host interactions, we analyzed serum metabolic profiles. Principal Component Analysis (PCA) revealed distinct metabolomic profiles between NF-PitNET patients and HCs, indicating clear, group-specific metabolic alterations (Figure 5A).

A detailed examination of serum metabolites showed significant differences between the groups. Hierarchical clustering analysis provided an intuitive visualization of metabolite expression patterns and sample relationships (Figure 5B). We identified 57 upregulated and 97 downregulated metabolites in the NF-PitNET group compared to HCs (Figure 5C). These differentially expressed metabolites were primarily involved in pathways such as ‘Phenylalanine, Tyrosine, and Tryptophan Metabolism’, the ‘Pentose Phosphate Pathway’, and ‘Alanine, Aspartate, and Glutamate Metabolism’ (Figure 5D).

**Figure 6:**
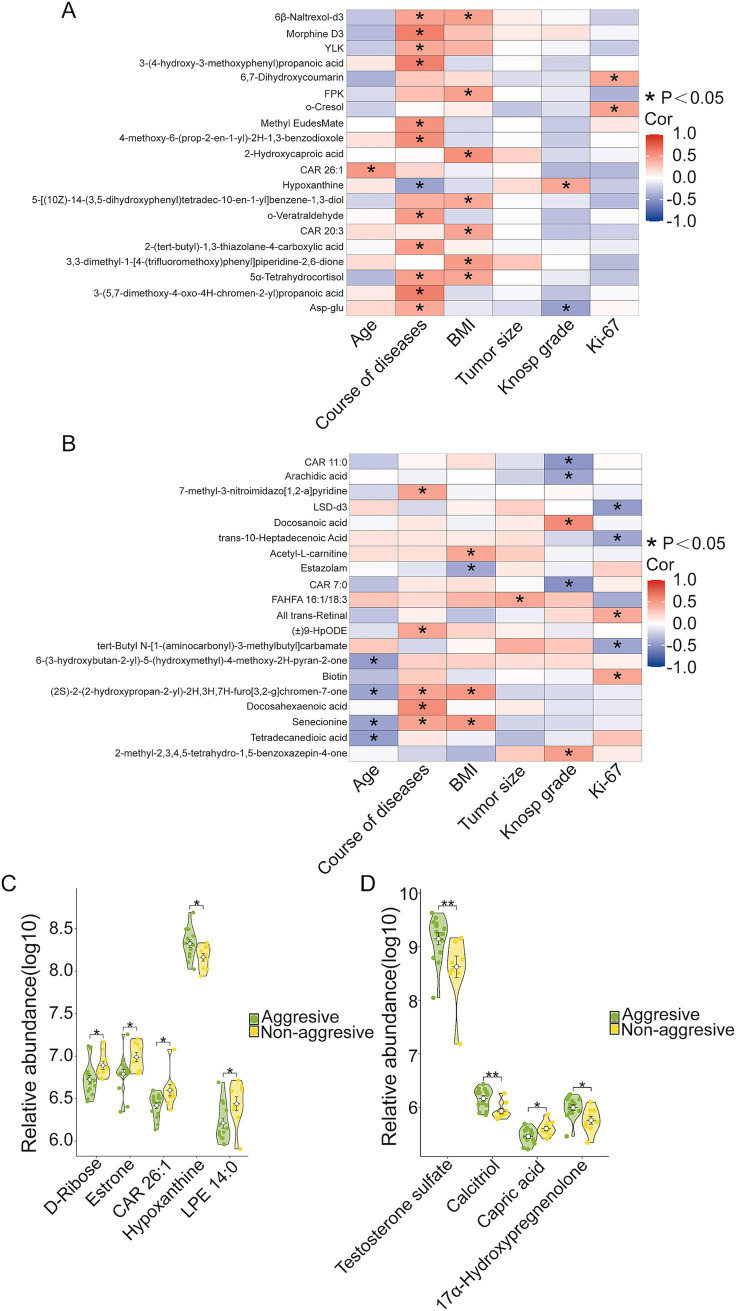
Correlation between differential metabolites and the severity of clinical symptoms. **(A)** Correlation between upregulated metabolites and the severity of clinical symptoms in NF-PitNETs. *p < 0.05. **(B)** Correlation between downregulated metabolites and the severity of clinical symptoms in NF-PitNETs. *p < 0.05. **(C)** Difference in the abundance of upregulated metabolites between aggressive and non-aggressive NF-PitNETs. *p < 0.05. **(D)** Difference in the abundance of downregulated metabolites between aggressive and non-aggressive NF-PitNETs. *p < 0.05, **p < 0.01.

Next, we analyzed the associations between differentially abundant metabolites and clinical phenotypes. As shown in Figures 6, 47 metabolites were linked to clinical features of the disease. Notably, three upregulated metabolites in the NF-PitNET group—6,7-Dihydroxycoumarin, o-Cresol, and Hypoxanthine—were positively correlated with indicators of disease severity, with 6,7-Dihydroxycoumarin and o-Cresol showing positive correlations with tumor Ki-67 expression, and Hypoxanthine correlating positively with tumor Knosp grade and aggression (Figures 6A,C). Among the downregulated metabolites, CAR 7:0, CAR 11:0, and Arachidic acid were negatively correlated with tumor Knosp grade. Additionally, LSD-d3, trans-10-Heptadecenoic acid, and tert-Butyl N-[1-(aminocarbonyl)-3-methylbutyl] carbamate showed negative correlations with tumor Ki-67 expression (Figure 6B), while Capric acid was negatively associated with tumor aggression (Figure 6D).

### 3.5 Multi-omics analysis reveals distinctions between NF-PitNETs and HCs

Our comprehensive analysis examined Spearman correlations between microbial taxa and serum metabolites (Supplementary Figure S8). Bacterial genera more abundant in NF-PitNET patients primarily showed positive correlations with upregulated metabolites and negative correlations with downregulated metabolites. In contrast, less abundant genera displayed negative correlations with upregulated metabolites and positive correlations with downregulated metabolites. This intricate network of interactions underscores the bidirectional relationship between the gut microbiota and serum metabolites in NF-PitNET patients.

**Figure 7:**
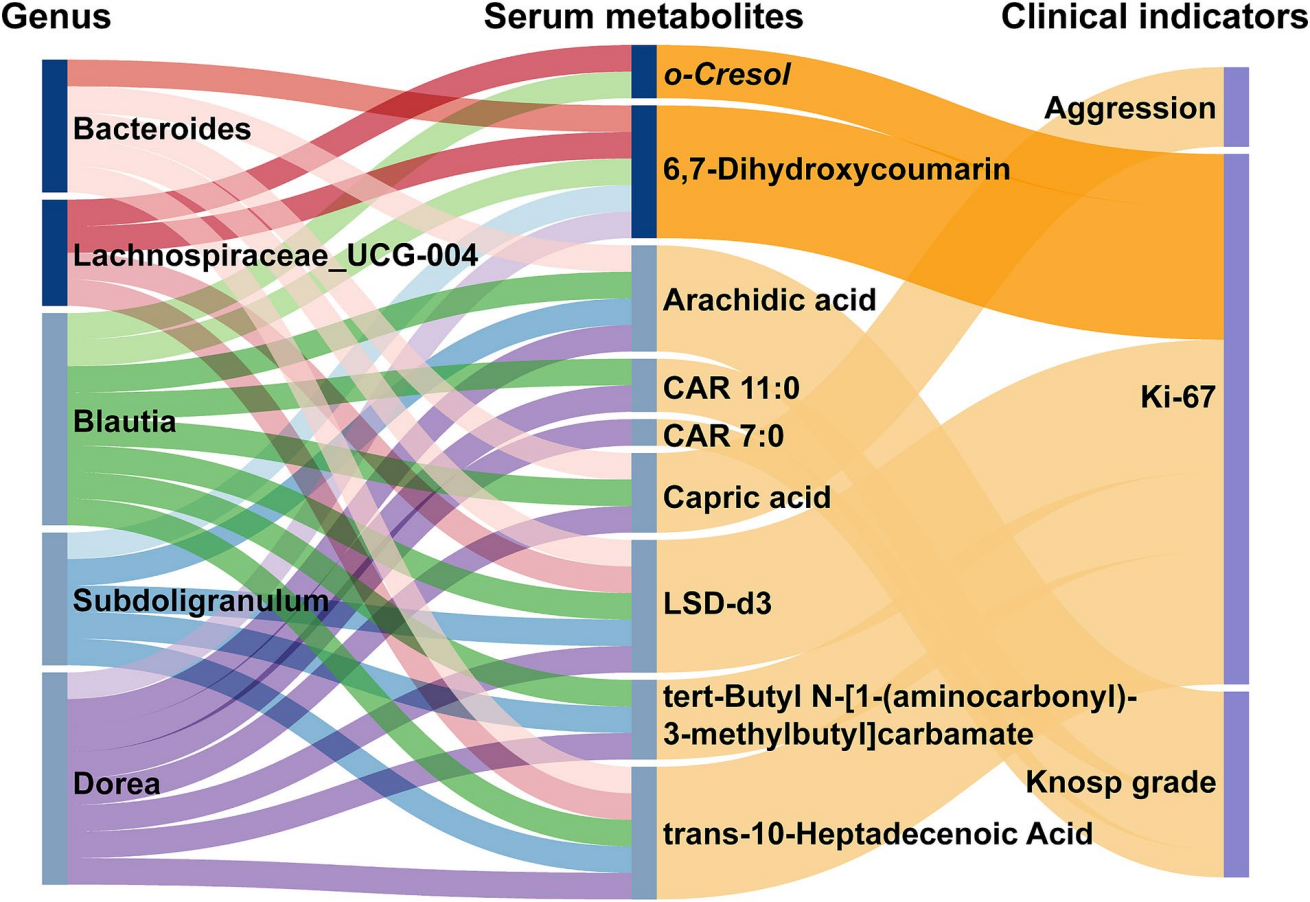
Sankey plot illustrating the interrelationship between gut flora, serum metabolic features, and major phenotypes. Dark lines represent positive correlations, while light lines represent negative correlations. In the first two columns of the plot, dark blue labels indicate NF-PitNETs-associated genera or metabolites, while light blue labels represent genera or metabolites negatively associated with NF-PitNETs.

The Sankey diagram in Figure 7 visualizes the complex correlations between bacterial flora, metabolites, and clinical indicators, illustrating the interrelationships among these variables. Five bacterial genera associated with NF-PitNETs were significantly (*p* < 0.05) linked to seven metabolites, which, in turn, were associated with disease severity indicators. We observed that upregulated genera in NF-PitNETs were positively correlated with adverse clinical phenotypes, either directly or via intermediary metabolites. Conversely, downregulated genera were negatively correlated with adverse clinical phenotypes, also either directly or through metabolites. Notably, Lachnospiraceae_UCG-004 was positively associated with elevated tumor Ki-67 expression, mediated by o-Cresol and 6,7-Dihydroxycoumarin, while Blautia and Subdoligranulum were negatively associated with Knosp grade, mediated by Arachidic Acid. These findings highlight specific microbial and metabolomic features linked to tumor characteristics, offering new insights into the mechanisms of disease progression and potential therapeutic targets.

## 4 Discussion

Emerging research has revealed intricate interactions between the gut microbiome and serum metabolome, which significantly influence cancer risk and progression. In particular, metabolites such as bile acids (BAs) and short-chain fatty acids (SCFAs), produced by intestinal microbiota, are critical mediators at the host-microorganism interface, exerting complex effects that can either promote or inhibit tumor development (Sepich-Poore et al., 2021; Stine et al., 2022; Marcobal et al., 2013). These findings underscore the importance of integrating microbiome and metabolome analyses to uncover new insights into cancer mechanisms.

In our study, we observed alterations in the intestinal microbiota and serum metabolite profiles of patients with NF-PitNETs, identifying potential biomarkers for diagnosis and treatment. We assessed the biodiversity of the intestinal flora between NF-PitNETs patients and healthy individuals. While alpha diversity showed no significant difference in the diversity and richness of the intestinal flora, beta diversity analyses revealed structural variations in the gut microbiota between the two groups, consistent with the findings of Hu et al. (2022). Using the BugBase database, we predicted an enrichment of potential pathogens in the NF-PitNETs group, while they were relatively absent in the healthy control group. These findings suggest that the intestinal flora may play a role in promoting the development of PitNETs.

Previous studies have found specific alterations in the gut microbiota of patients with NF-PitNETs. Notably, the relative abundance of *Oscillibacter* sp. 57_20 and *Fusobacterium mortiferum* is lower in patients with non-invasive NF-PitNETs, while *Clostridium innocuum* is upregulated in both invasive and non-invasive NF-PitNETs compared to healthy controls (Hu et al., 2022).

Our study revealed a significant increase in Bacteroides, Parabacteroides, Bilophila, and several genera within the Lachnospiraceae family in patients with NF-PitNETs compared to healthy controls. Bacteroides, for instance, has been associated with colorectal and breast cancers. Specifically, *Bacteroides fragilis* produces toxins that activate pathways such as NF-κB, SMO, Wnt/*β*-catenin, and Notch1, in addition to inducing reactive oxygen species (ROS) and DNA damage, all of which contribute to cancer progression (Parida et al., 2021; Cheng et al., 2020; Sears et al., 2014). Bilophila generates hydrogen sulfide (H₂S), a compound known to damage DNA, stimulate inflammation, and promote cellular proliferation in the colon. This effect is particularly pronounced in individuals with high-protein, high-fat diets, potentially increasing the risk of colorectal cancer development (Yazici et al., 2017). The downregulated microbiota observed in NF-PitNET patients in our study includes well-known protective intestinal flora that may contribute to tumor prevention and suppression through multiple mechanisms. For instance, Blautia and the *Eubacterium hallii* group produce short-chain fatty acids (SCFAs), particularly butyrate, which exhibits anti-inflammatory properties and inhibits cancer cell proliferation by modulating immune responses (Ye et al., 2023; Song et al., 2024). Additionally, Bifidobacterium has been shown to improve the efficacy of immune checkpoint inhibitors by strengthening the gut barrier and promoting anti-tumor immune responses (Sivan et al., 2015). Together, these bacteria foster a protective gut microbiome profile, reduce systemic inflammation, and provide metabolites that inhibit tumor growth while enhancing immune surveillance against cancer cells.

The differential expression of microbial genera linked to aggressive versus non-aggressive tumor phenotypes further underscores the role of specific microbiota in disease progression. For example, elevated Bacteroides in aggressive NF-PitNETs might reflect an environment favoring tumor invasion, supported by positive correlations with clinical indicators such as tumor size and Knosp grade. These observations suggest that microbiota-targeted therapies could represent a novel avenue for managing tumor aggressiveness in NF-PitNETs.

The serum metabolomic profiles of NF-PitNET patients displayed notable alterations, with differential expression of 154 metabolites predominantly linked to pathways involved in amino acid and carbohydrate metabolism. For instance, pathways such as “Phenylalanine, Tyrosine, and Tryptophan Metabolism” and the “Pentose Phosphate Pathway” are integral to cellular growth and immune regulation (TeSlaa et al., 2023; Liu et al., 2024; Lieu et al., 2020; Landis et al., 2018), and their dysregulation could support the proliferation and invasiveness of pituitary tumors. Upregulated metabolites, such as o-cresol and hypoxanthine, were positively associated with aggressive clinical characteristics. o-Cresol may induce cell membrane damage, trigger free radical reactions, alter glycolytic processes, or disrupt carcinogen metabolism, thereby promoting carcinogenesis (Yanysheva et al., 1993). Hypoxanthine plays a critical role in the nucleotide salvage pathway, enabling cells to recycle purine bases like hypoxanthine to generate essential nucleotides without relying on energy-intensive *de novo* synthesis. This pathway is particularly significant for cancer cells, which require large amounts of purines to support rapid proliferation (Shakartalla et al., 2024). Our multi-omics analysis revealed correlations between specific microbial genera and metabolites, further linking gut dysbiosis with NF-PitNET metabolic disturbances. The positive correlations observed between upregulated bacterial genera (e.g., Lachnospiraceae_UCG-004) and pro-tumor metabolites (e.g., o-Cresol) underscore the intricate interactions by which the gut microbiome could influence tumor behavior. In contrast, downregulated genera such as Blautia and Subdoligranulum, which were negatively correlated with tumor markers, may serve as protective factors through their associations with anti-inflammatory metabolites like Arachidic Acid. This interplay suggests that therapeutic strategies aimed at enhancing beneficial microbes or supplementing anti-inflammatory metabolites could potentially mitigate NF-PitNET progression.

While our findings provide new insights into the gut microbiota and serum metabolomic alterations in NF-PitNET patients, we acknowledge the potential impact of confounding factors. For instance, the *p*-values for sex (0.058) and BMI (0.055) are close to the significance threshold, indicating that these variables might influence the observed microbial and metabolic differences. We recognize the benefits of age-, sex-, and BMI-matched designs and will consider adopting this approach in future studies to improve comparability.

Furthermore, this study is exploratory in nature, and we acknowledge certain limitations. While 16S rRNA gene sequencing is a widely used method for microbiome analysis, it does not provide comprehensive genetic characterization. Additionally, our relatively small sample size and single-center data collection limit the generalizability of our findings. Larger, multi-center studies with more controlled experimental designs will be necessary to validate our observations and further explore the mechanisms underlying the microbiota-metabolome-tumor axis in NF-PitNETs.

Our findings shed light on the complex interactions between gut microbiota and host metabolism in NF-PitNET patients, offering insights into potential biomarkers for disease progression and therapeutic targets. Identifying microbial and metabolic profiles linked to tumor aggressiveness and metabolic reprogramming presents an opportunity for developing personalized treatment strategies. Modulating the gut microbiota and rebalancing key metabolites may offer a means to influence the tumor microenvironment, potentially reducing tumor growth and invasion.

## 5 Conclusion

This study highlights distinct gut microbiota and serum metabolite differences in NF-PitNET patients compared to healthy individuals. Key findings include an increase in inflammatory bacteria like Bacteroides and metabolites like o-Cresol, associated with aggressive tumor characteristics. The altered gut bacteria are linked with tumor-promoting metabolites, suggesting gut dysbiosis might play a role in NF-PitNET progression. While promising, further studies with larger sample sizes and more advanced sequencing are needed to confirm these potential biomarkers and therapeutic targets.

## Data Availability

The original contributions presented in the study are publicly available. This 16S rRNA sequencing data can be found here: https://www.ncbi.nlm.nih.gov/bioproject/PRJNA1231099/. The metabolomics data has been submitted to the MetaboLights data repository (https://www.ebi.ac.uk/metabolights/), accession number MTBLS12328.

## Footnotes

^1^ https://www.arb-silva.de/

## Supplementary material

Supplementary Figure S1 Gut microbiome diversity analysis. **(A)** Rarefaction curves. **(B)** Beta diversity analysis using weighted UniFrac, followed by ANOSIM.

Supplementary Figure S2 LDA combined with LEfSe. **(A)** Histogram of LDA scores, where the LDA score reflects the effect size and ranking of each differentially abundant taxon (LDA > 3.6). **(B)** Cladogram showing the phylogenetic distribution of microbiota associated with the HC and NF-PitNETs groups.

Supplementary Figure S3 LDA combined with LEfSe. Histogram of LDA scores, where the LDA score reflects the effect size and ranking of each differentially abundant genus (LDA > 3.0).

Supplementary Figure S4 Differences in bacterial genera between aggressive and non-aggressive NF-PitNETs. **(A)** Difference in the abundance of upregulated bacterial genera between aggressive and non-aggressive NF-PitNETs. **P* < 0.05, ns: not significant. **(B)** Difference in the abundance of downregulated bacterial genera between aggressive and non-aggressive NF-PitNETs. **P* < 0.05, ns: not significant.

Supplementary Figure S5 Differential pathways between the NF-PitNET and HC groups predicted by PICRUSt2.

Supplementary Figure S6 Diversity analysis of the gut microbiota. **(A)** The Shannon index and the Simpson index were used to estimate alpha diversity differences between the aggressive and non-aggressive groups. **(B)** Beta diversity analysis comparing the aggressive and non-aggressive groups.

Supplementary Figure S7 LDA combined with LEfSe. Histogram of LDA scores reflecting the effect size and rank of each differentially abundant genus between the aggressive and non-aggressive groups (LDA > 3.0).

Supplementary Figure S8 Integrated correlation analysis of microbes and metabolites. Heatmap depicting Spearman’s rank correlation analysis between differential gut microbiota and serum metabolites. *P < 0.05.

## Glossary

16S rRNA: 16S ribosomal RNA
Adonis: Permutational multivariate analysis of variance
Anosim: Analysis of similarities
BA: Bile acid
BFT: *Bacteroides fragilis* toxin
BMI: Body mass index
CV: Coefficient of variation
DCA: Deoxycholic acid
FDR: False discovery rate
GLM: Generalized linear model
HC: Healthy control
H₂S: Hydrogen sulfide
Ki67: A protein associated with cell proliferation
LC/MS: Liquid chromatography-mass spectrometry
LDA: Linear discriminant analysis
LEfSe: Linear discriminant analysis effect size
m/z: Mass-to-charge ratio
MRPP: Multi-response permutation procedure
NF-PitNET: Non-functional pituitary neuroendocrine tumor
OTU: Operational taxonomic unit
PA: Pituitary adenoma
P53: A tumor suppressor protein
PCoA: Principal coordinate analysis
PCA: Principal component analysis
QC: Quality control
QIIME: Quantitative insights into microbial ecology
R: Correlation coefficient in statistical analysis
*R* ^2^: Coefficient of determination
ROS: Reactive oxygen species
SASP: Senescence-associated secretory phenotype
SCFA: Short-chain fatty acids
UniFrac: A method used to measure the phylogenetic distance between microbial communities
