# Supplementary figures and images for: Exploring the gut microbiome and serum metabolome interplay in non-functioning pituitary neuroendocrine tumors
Source: Front Microbiol. 2025 Apr 1;16:1541683. doi: 10.3389/fmicb.2025.1541683 (PMC11997625; doi:10.3389/fmicb.2025.1541683)

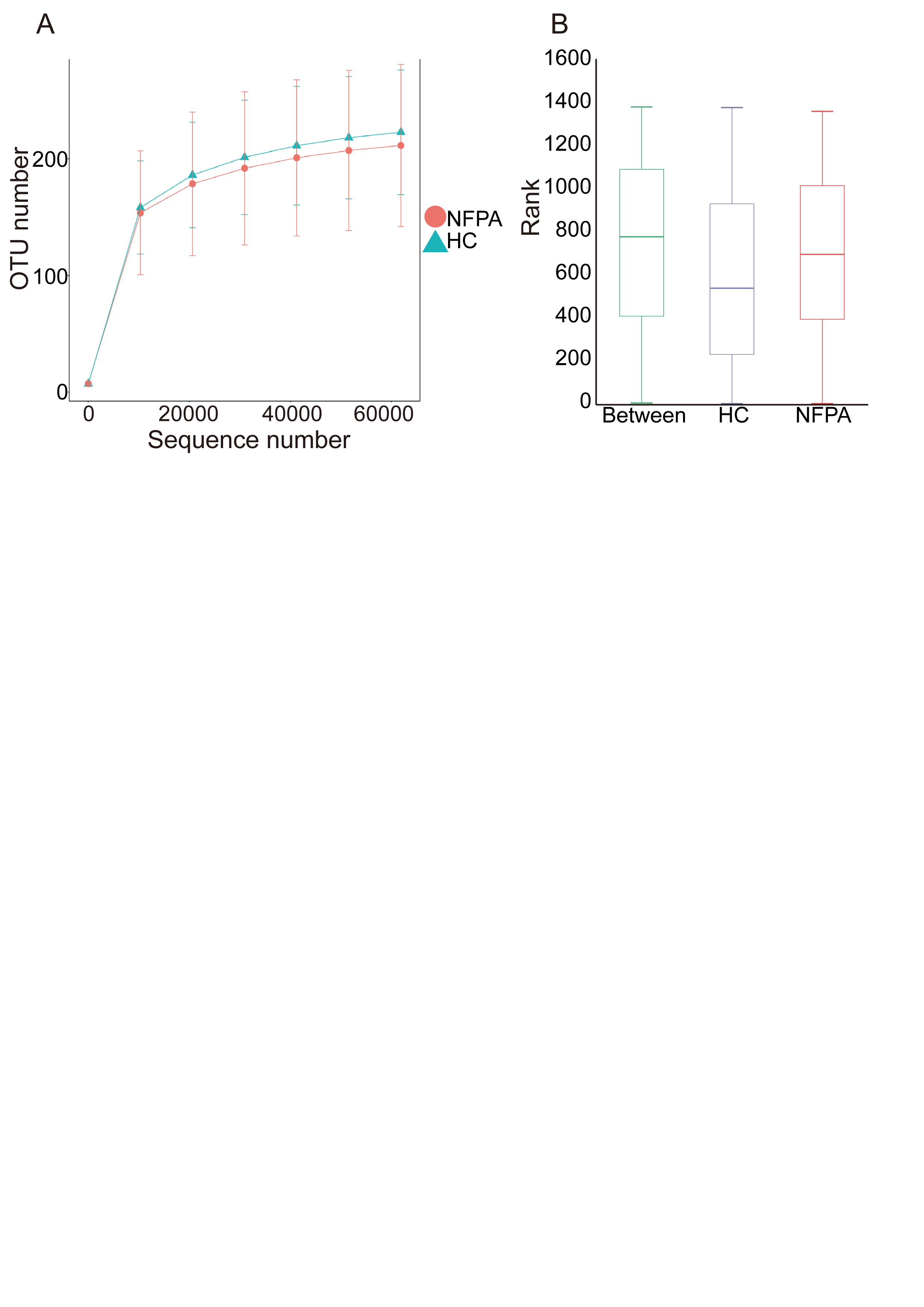

Supplement: Supplementary Figure S1 — Gut microbiome diversity analysis. (A) Rarefaction curves. (B) Beta diversity analysis using weighted UniFrac, followed by ANOSIM. [file Image_1.tif]

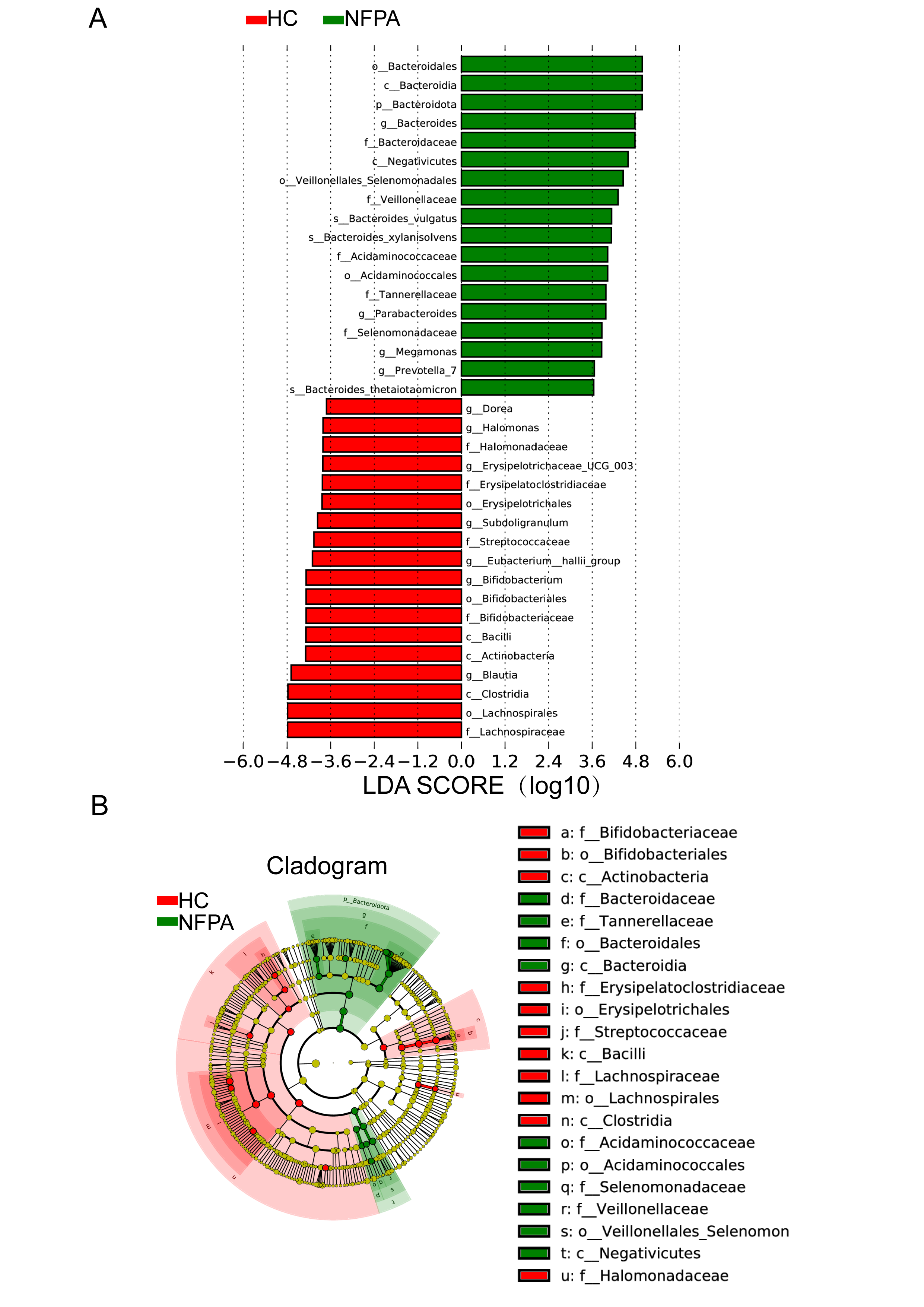

Supplement: Supplementary Figure S2 — LDA combined with LEfSe. (A) Histogram of LDA scores, where the LDA score reflects the effect size and ranking of each differentially abundant taxon (LDA > 3.6). (B) Cladogram showing the phylogenetic distribution of microbiota associated with the HC and NF-PitNETs groups. [file Image_2.tif]

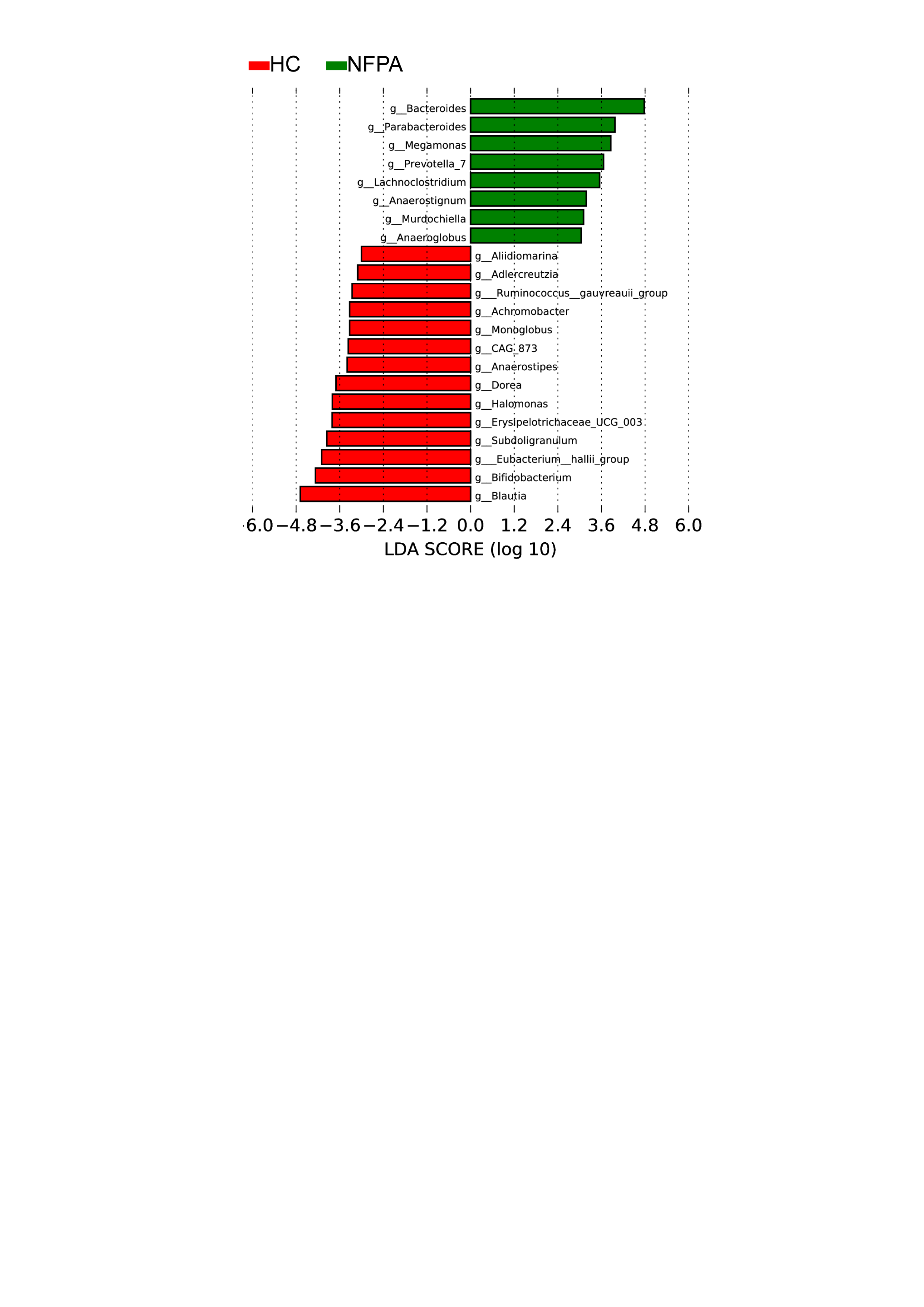

Supplement: Supplementary Figure S3 — LDA combined with LEfSe. Histogram of LDA scores, where the LDA score reflects the effect size and ranking of each differentially abundant genus (LDA > 3.0). [file Image_3.tif]

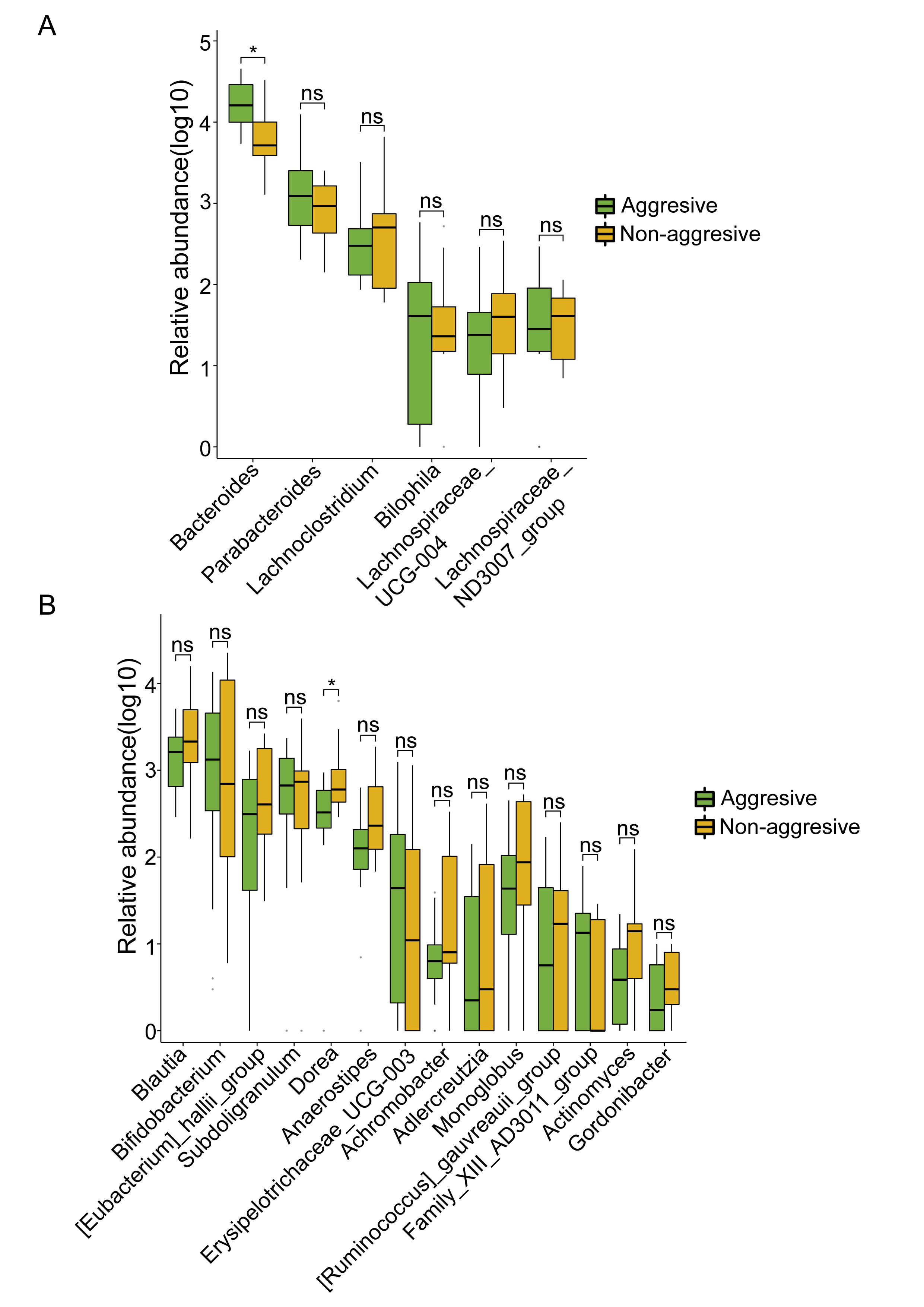

Supplement: Supplementary Figure S4 — Differences in bacterial genera between aggressive and non-aggressive NF-PitNETs. (A) Difference in the abundance of upregulated bacterial genera between aggressive and non-aggressive NF-PitNETs. *P < 0.05, ns: not significant. (B) Difference in the abundance of downregulated bacterial genera between aggressive and non-aggressive NF-PitNETs. *P < 0.05, ns: not significant. [file Image_4.tif]

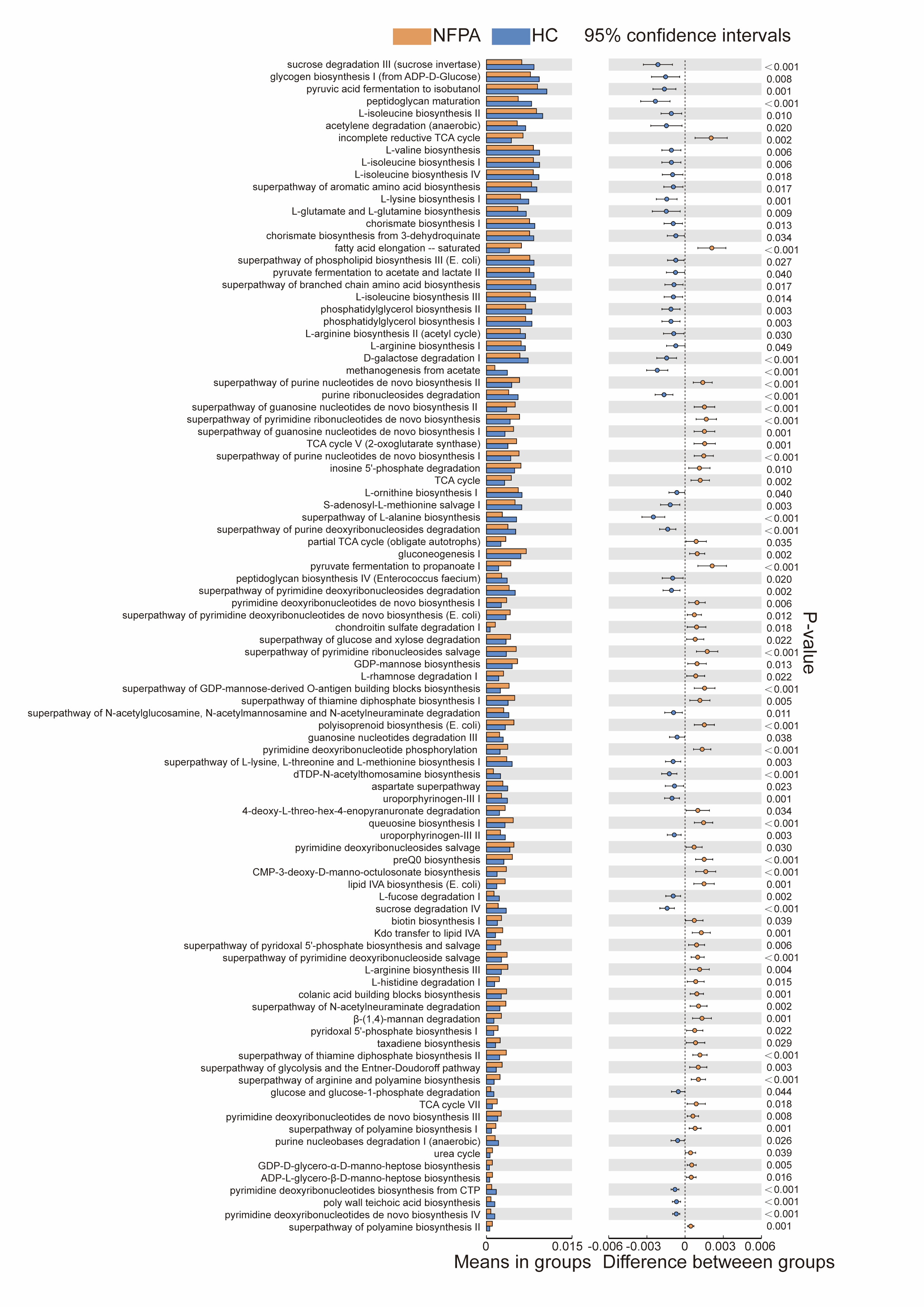

Supplement: Supplementary Figure S5 — Differential pathways between the NF-PitNET and HC groups predicted by PICRUSt2. [file Image_5.tif]

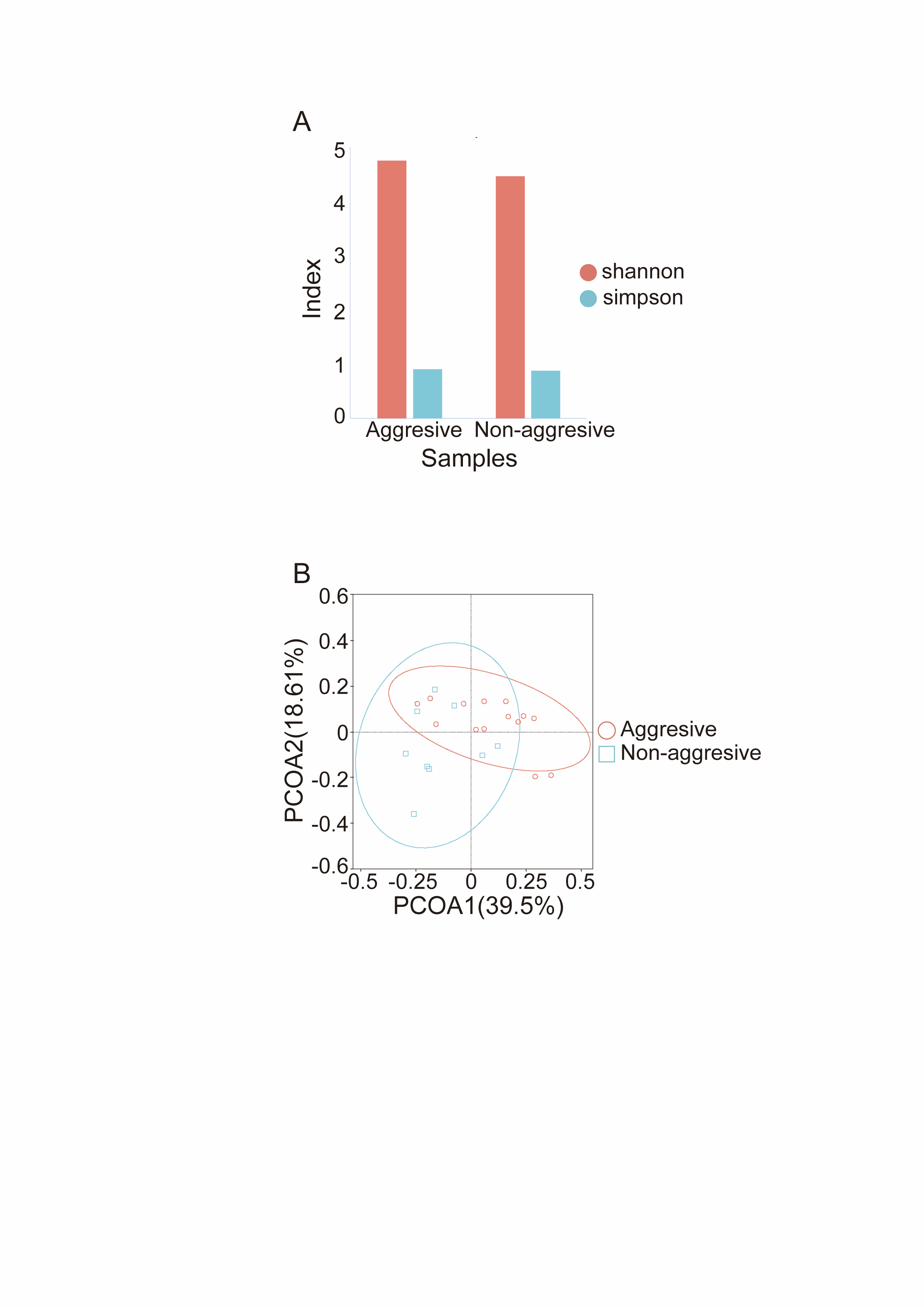

Supplement: Supplementary Figure S6 — Diversity analysis of the gut microbiota. (A) The Shannon index and the Simpson index were used to estimate alpha diversity differences between the aggressive and non-aggressive groups. (B) Beta diversity analysis comparing the aggressive and non-aggressive groups. [file Image_6.tif]

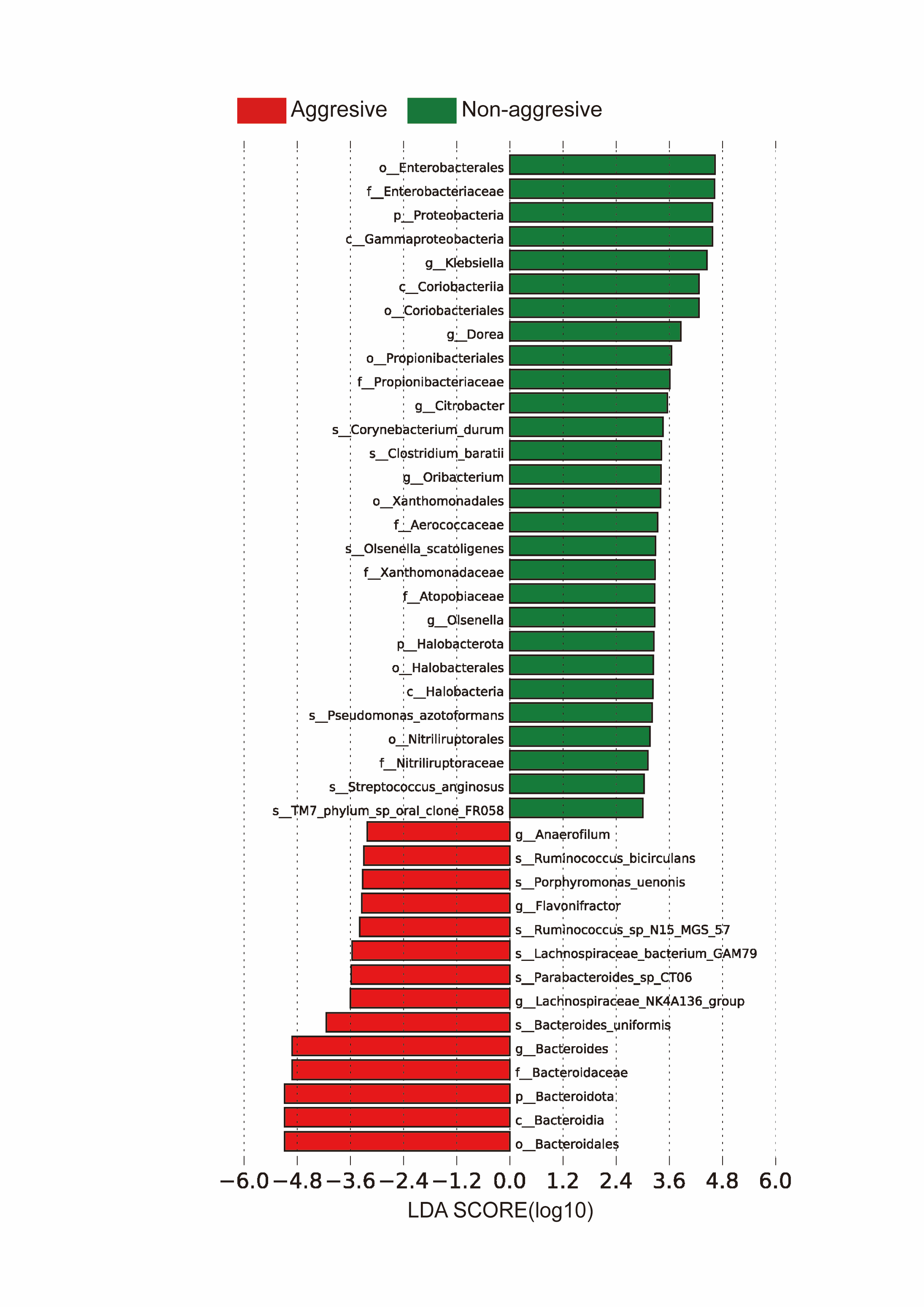

Supplement: Supplementary Figure S7 — LDA combined with LEfSe. Histogram of LDA scores reflecting the effect size and rank of each differentially abundant genus between the aggressive and non-aggressive groups (LDA > 3.0). [file Image_7.tif]

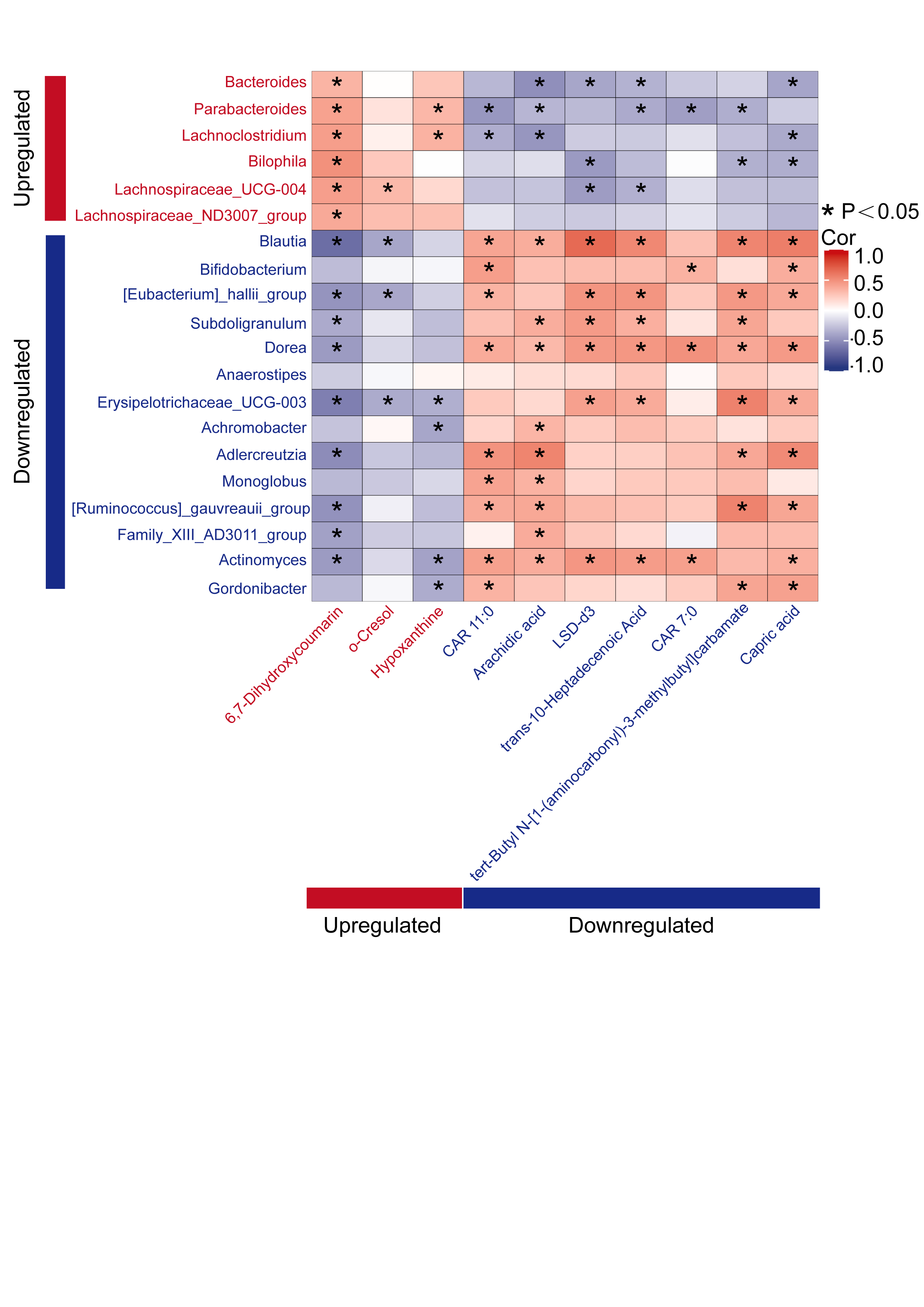

Supplement: Supplementary Figure S8 — Integrated correlation analysis of microbes and metabolites. Heatmap depicting Spearman’s rank correlation analysis between differential gut microbiota and serum metabolites. *P < 0.05. [file Image_8.tif]
